# Spatiotemporal Variations and Factors of Air Quality in Urban Central China during 2013–2015

**DOI:** 10.3390/ijerph17010229

**Published:** 2019-12-28

**Authors:** Mao Mao, Xiaolin Zhang, Yamei Shao, Yan Yin

**Affiliations:** Key Laboratory for Aerosol-Cloud-Precipitation of China Meteorological Administration, School of Atmospheric Physics, Nanjing University of Information Science & Technology, Nanjing 210044, China

**Keywords:** particulate matter, pollution sources, premature mortality, trace gas, PSCF/CWT

## Abstract

Spatiotemporal behaviors of particulate matter (PM_2.5_ and PM_10_) and trace gases (SO_2_, NO_2_, CO, and O_3_) in Hefei during the period from December 2013 to November 2015 are investigated. The mean annual PM_2.5_ (PM_10_) concentrations are 89.1 ± 59.4 µg/m^3^ (118.9 ± 66.8 µg/m^3^) and 61.6 ± 32.2 µg/m^3^ (91.3 ± 40.9 µg/m^3^) during 2014 and 2015, respectively, remarkably exceeding the Chinese Ambient Air Quality Standards (CAAQS) grade II. All trace gases basically meet the requirements though NO_2_ and O_3_ have a certain upward trend. Old districts have the highest pollution levels, followed by urban periphery sites and new districts. Severe haze pollution occurs in Hefei, with frequent exceedances in particulate matter with 178 (91) days in 2014 (2015). The abnormal PM_2.5_ concentrations in June 2014 attributed to agricultural biomass burning from moderate resolution imaging spectroradiometry (MODIS) wildfire maps and aerosol optical depth (AOD) analysis. PM_2.5_ is recognized as the major pollutant, and a longer interspecies relationship is found between PM_2.5_ and other criteria pollutants for episode days as compared to non-episode days. The air pollution in Hefei tends to be influenced by local primary emissions, secondary formation, and regional transport from adjacent cities and remote regions. Most areas of Anhui, southern Jiangsu, northern Zhejiang, and western Shandong are identified as the common high-potential source regions of PM_2.5_. Approximately 9.44 and 8.53 thousand premature mortalities are attributed to PM_2.5_ exposure in 2014 and 2015. The mortality benefits will be 32% (24%), 47% (41%), 70% (67%), and 85% (83%) of the total premature mortalities in 2014 (2015) when PM_2.5_ concentrations meet the CAAQS grade II, the World Health Organization (WHO) IT-2, IT-3, and Air Quality Guideline, respectively. Hence, joint pollution prevention and control measures need to be strengthened due to pollutant regional diffusion, and much higher health benefits could be achieved as the Hefei government adopts more stringent WHO guidelines for PM_2.5_.

## 1. Introduction

In recent decades, China is facing severe air pollution with the booming economic growth and dramatic increase in energy consumption [1,2]. Health burden attributable to particulate matter (PM) has become a matter of public concern [3,4,5,6]. About 1.3 million deaths each year in China were attributable to ambient PM_2.5_ (PM with an aerodynamic diameter less than 2.5 µm) exposure [7]. It is necessary for us to understand the characteristics, sources, and influencing factors of pollutants to alleviate air pollution for health sake.

The government has formulated and promulgated a series of standards, laws, and regulations to reduce air pollutant emissions and improve environment quality [8]. The Chinese Ministry of Environmental Protection has issued air pollution index (API) starting from June 2000 in major cities, and the pollutants involve in the evaluation including 24 h average concentrations of ground measured sulfur dioxide (SO_2_), nitrogen dioxide (NO_2_), and PM_10_ (PM with an aerodynamic diameter less than 10 µm) (http://datacenter.mep.gov.cn/). The index is published only once a day, unable to accurately assess air quality level. Subsequently, PM_2.5_, carbon monoxide (CO), and 8 h peak ozone (O_3_ 8h) as the routine monitoring pollutants are adopted into the newly revised Chinese Ambient Air Quality Standards (CAAQS, GB3095-2012) by the China State Council on February 2012 [9]. The real-time hourly average concentration of six criteria of air pollutants and associated air quality index (AQI) are released for public scrutiny and scientific research [10].

Numerous studies of air pollution are implemented in China, especially for some representative megalopolis, such as Beijing, Shanghai, Guangzhou, Nanjing, and abundant observation data and research results have been obtained [11,12,13,14,15,16,17]. On the contrary, in Hefei (30.57–32.32° N, 116.41–117.58° E), the capital city of Anhui Province, central China, only a few sole researches of air pollution characteristics have been sporadically conducted to date. Huang et al. analyzed daily PM_10_ and visibility data in 2001–2012 to investigate the air quality status as well as the long-term pollution trends in the city of Hefei [18]. Zhang et al. evaluated the temporal variation of black carbon aerosols with year-round measurements [19,20]. Furthermore, the aerosol radiative characteristics were also evaluated to classify severe brown haze episodes in Hefei [21]. Hong et al. examined the influence of haze on the chemistry of atmospheric mercury on haze days [22]. Acute air pollution exposure was significantly associated with cardiovascular morbidity risk [23]. Hu et al. investigated particle-associated polycyclic aromatic hydrocarbons and assessed the health risks with the index of toxic equivalent concentration [24]. Heavy metal elements (S, Zn, Cu, and Pb) took up a large proportion of the overall PM concentrations in Hefei [25]. The rapid economic development has caused unoptimistic air condition in Hefei during recent years.

To the best of our knowledge, related studies of six criteria pollutants and corresponding influence factors in Hefei are still lacking so far. To fill up the research gap, the major objectives of this work are organized as follows: (1) to reveal the characteristics of six criteria pollutants, including NO_2_, SO_2_, CO, O_3_, PM_2.5_, and PM_10_ and their annual, seasonal, monthly, and diurnal variations. (2) to investigate interspecies relationship and the relationship between pollutants and meteorological elements. (3) to identify the transport pathway and regional potential source contributions of PM based on the cluster analysis of backward trajectories, the potential source contribution function (PSCF) and concentration-weighted trajectory (CWT) models. (4) to estimate premature mortality (ΔMort) due to PM_2.5_ exposure and potential mortality benefits in scenarios where PM_2.5_ concentrations in Hefei meet the CAAQS grade II, the World Health Organization (WHO) interim target (IT)-2, IT-3, and Air Quality Guideline (AQG) with integrated exposure–response (IER) function from December 2013 to November 2015. The new findings will be helpful to understand the current situation of environmental quality, enhance the environmental awareness of the general public, and enable new perspectives on how to formulate effective incentives to retrofit air pollution control strategy in central China if necessarily in time.

## 2. Data and Methodology

**Figure 1:**
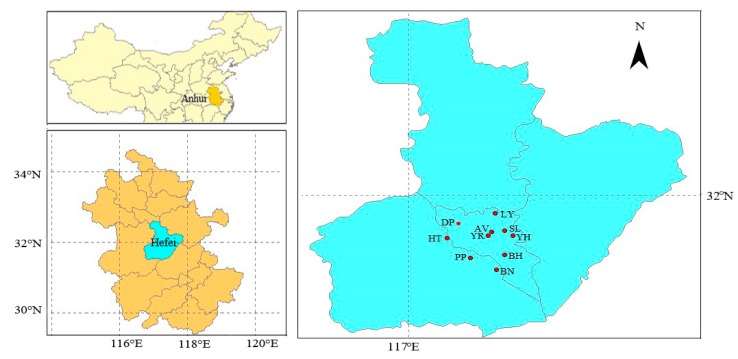
The location of Anhui Province (up-left), Hefei (down-left), and 10 air quality stations of Hefei (right).

Hefei is an inland medium-sized city situated in the west of the Yangtze River Delta (YRD). According to the 2015 Statistical Bulletin of Hefei, it has a permanent population of approximately 7.8 million, an occurrence of 1.2 million vehicles, and a gross domestic product (GDP) of 566.0 billion yuan. It is categorized as being in the transitional area between the temperate and subtropical zones with hot humid summer and cold dry winter. The real-time hourly average concentrations of six criteria pollutants from December 2013 to November 2015 in Hefei were downloaded from Chinese Environmental Protection Bureau (http://www.cnemc.cn). Two-year monitoring data are employed in this study, and the period from December 2013 to November 2014 represents the year 2014 while the period from December 2014 to November 2015 represents the year 2015. Figure 1 illustrates the location of Hefei and air quality monitoring stations. The ten monitoring sites are: Luyang District (LD; 31.94° N, 117.27° E), Dongpu Reservoir (DR, 31.91° N, 117.16° E), Sanlijie (SLJ, 31.88° N, 117.31° E), Amber Villa (AV, 31.87° N, 117.26° E), Yangtze River Road (YRR, 31.86° N, 117.25° E), Yaohai District (YD, 31.86° N, 117.34° E), High-tech Zone (HT, 31.85° N, 117.12° E), Baohe District (BD, 31.80° N, 117.30° E), Pearl Plaza (PP, 31.78° N, 117.20° E), and Binhu New Zone (BN, 31.74° N, 117.28° E). DR is a large-scale reservoir used for flood control with no nearby emission sources, representing background levels for the region. In this study, the 8 h, 24 h, or daily, monthly, seasonal, and annual concentrations of air pollutants were calculated by averaging the hourly data from all of the monitoring stations. The surface meteorological observations were obtained from Hefei Meteorological Bureau.

To determine the direction where the airborne masses reached the central location of Hefei (31.86° N, 117.25° E) qualitatively, 72 h backward trajectories were computed by using the Hybrid Single Particle Lagrange Integrated Trajectory (HYSPLIT)-4 model [26,27]. The PSCF model combines the results of backward-trajectory (8 times per day, 0:00, 3:00, 6:00, 9:00, 12:00, 15:00, 18:00, and 21:00 UTC) for reflecting the proportion of polluted trajectories. The CWT model is applied to weight trajectories with related PM_2.5_ concentrations for identifying the relative contribution of different source areas [28,29]. Areas with high PSCF/CWT values are supposed to be potential geographic origins of PM_2.5_. IER model is utilized to analyze the impact of long-term outdoor PM_2.5_ exposure on the ΔMort [30,31]. The cumulative deaths caused by stroke (STR, including ischemic and hemorrhagic stroke), ischemic heart disease (IHD), lung cancer (LC), and chronic obstructive pulmonary disease (COPD) are considered as the total number of ΔMort. For the detailed ‘Date and Methodology’ see the Supporting Information.

## 3. Results and Discussion

### 3.1. Annual Variation

The annual mean PM_2.5_ (PM_10_) concentrations are 89.1 ± 59.4 µg/m^3^ (118.9 ± 66.8 µg/m^3^) and 61.6 ± 32.2 µg/m^3^ (91.3 ± 40.9 µg/m^3^) during 2014 and 2015, respectively (Table 1), all of which remarkably exceed the CAAQS grade II (PM_2.5_ of 35 µg/m^3^ and PM_10_ of 70 µg/m^3^ for annual average) and WHO guideline (PM_2.5_ of 10 µg/m^3^ and PM_10_ of 20 µg/m^3^ for annual average) [32]. The standard deviation in 2014 is uniformity higher than that in 2015, which may be attributed to heavy atmospheric pollution during the period of the former. In 2008, the average annual PM_10_ concentration is 126 µg/m^3^, which is more serious than statistics in this work [18]. Compared to PM_2.5_ levels in the key cities of YRD, the mean concentration of PM_2.5_ in Hefei from December 2013 to November 2015 is higher than those in shanghai (52 µg/m^3^), Nanjing (65 µg/m^3^), Suzhou (62 µg/m^3^), Wuxi (64 µg/m^3^), Hangzhou (61 µg/m^3^), Ningbo (45 µg/m^3^), and Wuhu (62 µg/m^3^) [33]. Additionally, the annual mean concentration of PM_2.5_ in Hefei is lower than or similar with those in Beijing (83 µg/m^3^), Wuhan (76 µg/m^3^), Tianjin (77 µg/m^3^), and Jinan (88 µg/m^3^) while much higher than most coastal cities of China, including Dalian (50 µg/m^3^), Qingdao (55 µg/m^3^), Guangzhou (44 µg/m^3^), Xiamen (33 µg/m^3^), and Shenzhen (33 µg/m^3^) in China [33]. Concerning gaseous pollution, average annual SO_2_, NO_2_, CO, and O_3_ concentrations are 30.2 (17.3), 28.2 (31.0), 1.1 × 10^3^ (1.0 × 10^3^), and 50.4 (64.8) µg/m^3^ in Hefei during 2014 (2015), respectively. All trace gases basically meet CAAQS Grade II, further legislation and laws should be strengthened to restrain PM concentrations.

### 3.2. Seasonal and Monthly Variations

To better study the temporal changes of pollutants, the corresponding data are divided into four seasons. In general, the mean concentrations of pollutants except O_3_ exhibited consistent seasonal variations, with similar magnitude in spring and autumn, lower concentrations in summer, and higher values occurring in winter (Table 1).

**Figure 2:**
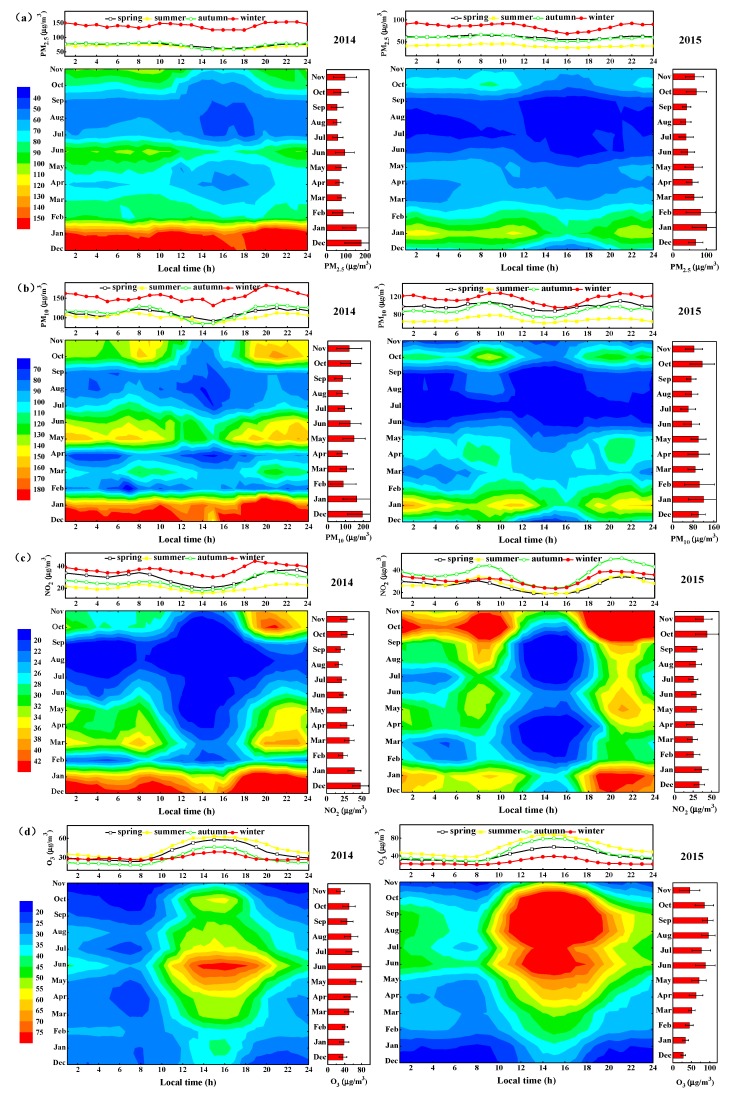
Diurnal variations of PM_2.5_ (**a**), PM_10_ (**b**), NO_2_ (**c**), and O_3_ (**d**).

In winter, high concentrations of pollutants are attributable to the extra primary emissions and secondary formation of PM_2.5_ as the coal burning for power generation during this season. Moreover, the variations in pollution concentrations are affected by meteorological factors. Unfavorable diffusion conditions such as lower rainfall, more frequent calm winds, and the prevalence of cold high-pressure system induce pollutant accumulation in a shallow layer with descending air motions [34,35]. Especially, December (179.6 ± 85.9 µg/m^3^ for PM_2.5_ and 195.0 ± 84.3 µg/m^3^ for PM_10_) and January (101.6 ± 43.9 µg/m^3^ for PM_2.5_ and 120.9 ± 59.2 µg/m^3^ for PM_10_) are the ‘worst’ months for PM concentrations in 2014 and 2015, respectively (Figure 2a,b). In addition, prevailing winds carry air pollutants from more serious areas to the sampling site, resulting in air quality degradation in winter.

**Figure 3:**
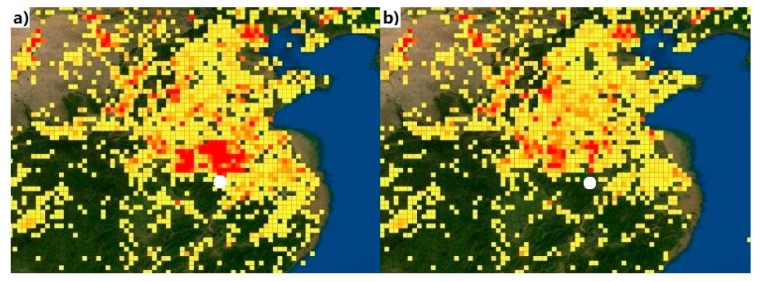
True color images derived from moderate resolution imaging spectroradiometry (MODIS) fires counts in June 2014 (**a**) and June 2015 (**b**). The white solid circle denotes the city of Hefei.

In summer, the corresponding precipitation accounts for nearly 50% of the annual precipitation (Table 2), and the increased mixed layer height and wet scavenging are conducive to pollutant dispersion. Nevertheless, the abnormal concentrations of PM_2.5_ are seen in June 2014 (Figure 2a), presumably caused by local straw burning. The information of dense fire points observed based on moderate resolution imaging spectroradiometry (MODIS) satellite [36] in Hefei and its surroundings could favor this viewpoint (Figure 3). As a big agricultural province, large-scale straw incineration is very common in rural areas of Anhui during summer and autumn. Numerous PM and gaseous contaminants are produced by incomplete combustion of straw residue in the open fields. After a series of supportive policies bound up with straw burning ban such as mechanization of straw chopping and returning to field are introduced by policy-makers, the air quality of Hefei improves obviously in June 2015. August (54.1 ± 20.2 µg/m^3^ for PM_2.5_ and 82.8 ± 31.5 µg/m^3^ for PM_10_ in 2014; 39.1 ± 15.2 µg/m^3^ for PM_2.5_ and 74.2 ± 24.3 µg/m^3^ for PM_10_ in 2015) is the ‘cleanest’ month for PM concentrations for both 2014 and 2015 identically.

**Figure 4:**
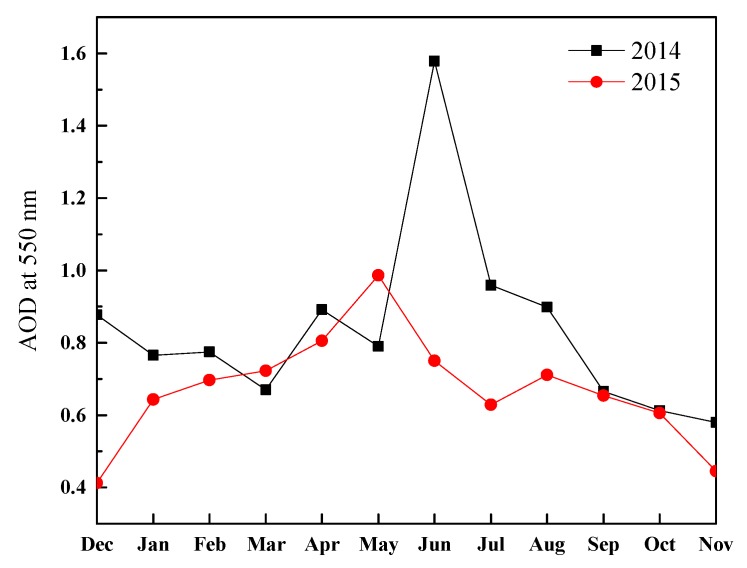
Monthly mean aerosol optical depth (AOD) map of area-averaged time series over urban Hefei (region: 116.5–117.5° E, 31–32° N) derived from MODIS-Terra data in 2014 and 2015.

Monthly average aerosol optical depth (AOD) values got from the Giovanni maps of the MODIS satellite during 2014 and 2015 are exhibited in Figure 4. The mean AOD in June 2014 has a maximum value of 1.58, probably due to summertime agricultural straw combustion, as opposed to the obviously low mean value of 0.75 in June 2015. No significant correlation between PM and AOD values are observed, and the following reasons can explicate the poor association. The AOD has a bearing on extinction properties of aerosol particles. Some AOD data may not be retrieved attributed to limitations such as orbit gaps, cloud masks, and sun-glint areas. The AOD describes the aerosol columnar integral over the entire atmosphere column and is averaged over some territories, whereas the PM data are gained at a few ground-based monitoring sites, describing local aerosol properties near the surface.

The monthly variations of NO_2_, SO_2_, and CO present a U-shaped pattern with pronounced maximums appearing in December or January (Figure 2c and Supplementary Figure S1). Similar reasons to PM could explicate these monthly patterns which are a combined effect of enhanced pollutant discharge and relatively stable synoptic conditions in winter. Last but not least, a stronger photochemistry reaction can remove the gases quickly from the atmosphere whereas it is least active and decelerates the transformation of primary gaseous pollutants during cold months [13,37]. Compared to that in 2014, the concentrations of PM_2.5_, PM_10_, SO_2_, and CO in 2015 exhibits a marked decline in almost four seasons owing to a series of policies released by local government, while NO_2_ concentration in the autumn of 2015 shows the abnormal increase. NO_2_ emissions primarily come from traffic vehicles, power plants, and industries with energy exhaust, and vehicles account for the most [38,39]. The number of motor vehicles is growing quickly as an economic flourish in Hefei. Additionally, the prevailing wind direction during this period is northeast. Pollutants are easily carried to the local area with the airflow movement, resulting in high amplitudes in autumn.

The O_3_ concentrations exhibit the opposite trends with inverted U-shape (Figure 2d). Its concentration begins to increase gradually from January and demonstrates a downward trend after reaching a maximum in June (79.6 ± 22.8 µg/m^3^ in 2014) or August (96.3 ± 18.1 µg/m^3^ in 2015), and the minimum of the whole year is in November (30.0 ± 9.7 µg/m^3^ in 2014) or December (28.2 ± 5.9 µg/m^3^ in 2015). Temperature directly influences O_3_ production by interfering with the photochemical reaction speed and the volatile organic compounds (VOCs) emissions [40,41]. The local emissions of precursors (NO_x_, VOC, and CO) also have an important effect on the regulation of O_3_ variability. Besides, O_3_ is also easily formed under the conditions of intense solar radiation induced by low PM concentrations [42,43]. Thus, by comparing with the O_3_ concentrations in 2014, the O_3_ in autumn and summer aggravates notably with 79.3% and 36.2% in 2015, presumably due to the stronger NO_2_ emissions and/or relative mitigation of PM release.

### 3.3. Diurnal Variations

The diurnal variations of pollutants are of great significance to understand the impact of potential emission sources and meteorological parameters related to domestic cooking, traffic, and industrialization in urban regions. The concentrations of PM_2.5_, PM_10_, NO_2_, and CO generally display a flat “M” pattern, with two peaks and two valleys (Figure 2 and Supplementary Figure S1). The first peak occurs around approximately in the rush hours before noon (9:00–11:00) and the second peak occurs at night (20:00–24:00) aggravated by the increased inter-cities vehicular traffic from freightage that is managed and controlled in daytime, suggesting the emissions of transportation are essential to the formation and accumulation of pollutants. For the morning peak, the spring, summer and fall are earlier than winter whereas for the evening peak the winter is earlier. The peaks appear at night during winter, especially for PM, are more pronounced than other seasons which may be explained by outset of heating (i.e., coal combustion) and relative stability weather. Correspondingly, the first valley occurs in the early morning (5:00–7:00) with less anthropogenic activity and pollutant deposition. The second lower valley occurs in the late afternoon (16:00–18:00) mainly because of the relatively high low planetary boundary layer (PBL) height and other meteorological conditions, which is conducive to air pollutant dispersion.

The O_3_ distribution demonstrates a unimodal distribution, and its concentrations generally start to increase at 7:00 and appear as a distinct peak at afternoon approximately 15:00 (Figure 2d). As the sun sets, with the decrease of the temperature and strength of solar radiation, the O_3_ concentrations cut down as well, especially at night in the cold season. The discrepancy of criteria pollutants with exception of NO_2_ and O_3_ among four seasons is more significant during 2014. Notably, the winter varying curves of PM_2.5_, PM_10_, CO, and SO_2_ are far above the curves for the other seasons during 2014 whereas the curves during spring, summer, and autumn are relatively close, indicating that winter pollution is particularly serious. Concerning the year 2015, the concentrations of PM_2.5_, PM_10_, CO, and SO_2_ reduce and the seasonal curves exhibit dispersedly distribution. The NO_2_ and O_3_ synchronously enhance during 2015 reflecting local anthropogenic emissions or regional transportation effects.

**Figure 5:**
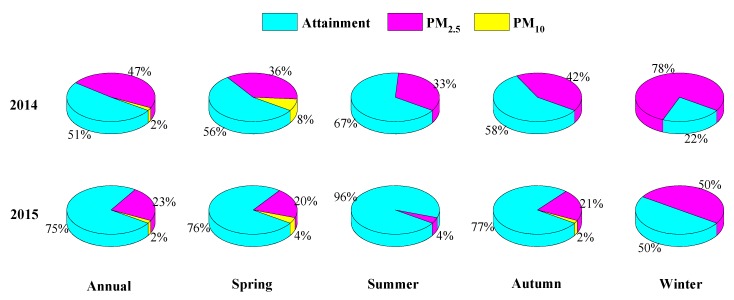
The fractions of major pollutants during 2014 and 2015.

To better understand the contribution of various components to ambient pollution, the proportions of major pollutants, defined as the pollutant with the largest AQI when AQI > 100 are presented in Figure 5. On the annual basis, PM_2.5_ is the dominant major pollutant and occupies about half of 2014 and a quarter of 2015. PM_10_ occasionally acts as the major pollutants on a small number of days with only 6–7 days. Astoundingly, no gas species are found as major pollutants during the studied period. A non-attainment day refers to the day with any pollutant concentration outstripping CAAQS grade II of concentration limits for 24 h average (AQI > 100). Hefei suffers severe air pollution with 178 days of exceedance during 2014, and the polluted proportion totals 49%. For 2015, air quality gets better with 91 days of exceedance and the substandard ratio of about 25%. A comprehensive analysis of the air quality evaluation on a seasonal basis is also made, and the non-attainment percentage is in the order of Winter (2014: 78%; 2015: 50%) > Spring (2014: 44%; 2015: 24%) > Autumn (2014: 42%; 2015: 23%) > Summer (2014: 33%; 2015: 4%). In contrary to trivial gas pollution, PM, especially PM_2.5_, is the main factor causing air pollution in Hefei.

### 3.4. Spatial Distribution

The detailed average ambient pollutant concentrations at different sampling sites during 2015 are summarized in Table 3. The highest mean daily PM concentrations are observed at YR and LY. Meanwhile, PP and DP have the highest O_3_ levels, SL has the highest NO_2_ concentration, and SO_2_ and CO concentrations at ten sites are similar. The different monitoring sites exhibit obvious spatial differences related much to their emission sources. PM_2.5_ congruously exceeds the CAAQS grade II more frequently than the other pollutants, implying air pollution in this region is primarily caused by a high level of PM_2.5_.

Generally, the sites clustered in old districts (YR, YH, SL, and AV) have the highest pollution levels, followed by urban periphery sites (LY, DP, BH, and PP), while new districts (HT and BN) have the cleanest air. The reasons for this phenomenon include the greater artificial release of PM caused by the larger population density and more frequent human activities such as vehicles and catering. Furthermore, the southern part of Hefei (BN, BH, PP, and HT) is located very close the lake district of Lake Chaohu, where the air is relatively moist and anthropogenic emissions are relatively small. On the contrary, the northern part of Hefei is near coal cities such as Huainan. As a result, the PM concentrations in the north sites are higher than those in the south.

In addition to the high concentration of PM in the old districts, serious pollution is also observed at LY on the urban periphery. The government supports and encourages the development of Luyang Industrial Park, focusing on the boom of modern printing, heat and power equipment, building materials, as well as agricultural by-products processing industries. Both distinct urban air pollution and straw burning in nearby suburb affect air quality in LY. PM pollution is alleviated at DP, the background site of Hefei. Botanical Garden is located to the east of DP site, and DP is surrounded by reservoirs using for flood control with no nearby emission sources, possessing favorable topographical conditions for atmospheric dispersion. Higher O_3_ concentrations occur at DP, attributed to biogenic VOC emissions. Additionally, the seasonal variation tendency of pollutant concentrations at ten sites shows high consistency (no shown), implying the regional characteristic of six pollutants in the atmosphere at Hefei to some extent.

### 3.5. Air Pollutants on Episode and Non-Episode Days

PM_2.5_ pollution in Hefei is serious with about 49% and 25% of the daily concentrations exceed the diurnal limit during 2014 and 2015, respectively. Herein, the days are classified into episode days (PM_2.5_ > 75 μg/m^3^) and non-episode days. The PM_2.5_/PM_10_ ratio and PM_2.5_/CO ratio are calculated to characterize the PM pollution further. These ratios can not only provide insights into characteristics of particle pollution but also have been used to reveal the sources of PM [35,44]. Both PM_2.5_/PM_10_ ratios and PM_2.5_/CO ratios on episode days (PM_2.5_/PM_10_: 0.74 for 2014 and 2015; PM_2.5_/CO: 0.095 for 2014 and 0.084 for 2015, respectively) are obviously higher than that on non-episode days (PM_2.5_/PM_10_: 0.62 for 2014 and 0.61 for 2015; PM_2.5_/CO: 0.062 for 2014 and 0.052 for 2015, respectively). The interspecies relationship between the PM_2.5_ and PM_10_ is also examined in terms of Pearson correlations (R) analysis (Table 4), and the results exhibit that the value is 0.81 during episode days, significantly higher than that in non-episode period (0.68), further demonstrating PM_2.5_ accounts for large fractions of PM_10_ and the differences between the two sources are small in episode pollution period. The CO is a key molecular marker of the intensity of anthropogenic burning [45,46,47]. Diurnal CO concentrations are 1.41 mg/m^3^ and 1.33 mg/m^3^ during the episode days of 2014 and 2015, obviously higher than those of the non-episode days (0.81 mg/m^3^ for 2014 and 0.89 mg/m^3^ for 2015) as well. Simultaneously, more remarkable positive correlations are found between PM_2.5_ and CO for episode days (0.82 for episode days and 0.57 for non-episode days), implying that CO emission process is accompanied by the emission of PM_2.5_. Moreover, stronger correlations are found between PM_2.5_ and NO_2_/SO_2_ for episode days (Table 4). High ratios in PM_2.5_/PM_10_ and PM_2.5_/CO during haze episode days are attributed to the formation of secondary aerosols by the oxidation of gaseous pollutants (SO_2_, NO_2_, and CO). The fluctuations for seasonal variations of PM_2.5_/PM_10_ and PM_2.5_/CO are mainly related to anthropogenic sources and atmospheric conditions (Supplementary Figure S2).

Besides the direct primary emissions and secondary formation of PM, the meteorological conditions during episode days in Hefei are also playing an important effect on accelerating the aggregation of PM_2.5_ (Table 2 and Table 4). A negative relationship is found between temperature and PM and gaseous pollutants except O_3._ Based on the analysis of the temperature pattern, the episode days occur more often during the lowest temperature. For example, the highest frequency of episode days is up to 53% with T < 7 ^o^C, whereas the corresponding data is about 28% as T > 25 ^o^C during the entire period. Relative humidity, accumulated rainfall, and wind speed are higher in non-episode days, and all exhibit more strongly reverse correlation with PM as compared to that in episode days.

### 3.6. Pollutants Source Analysis Based on HYSPLIT, PSCF, and CWT

**Figure 6:**
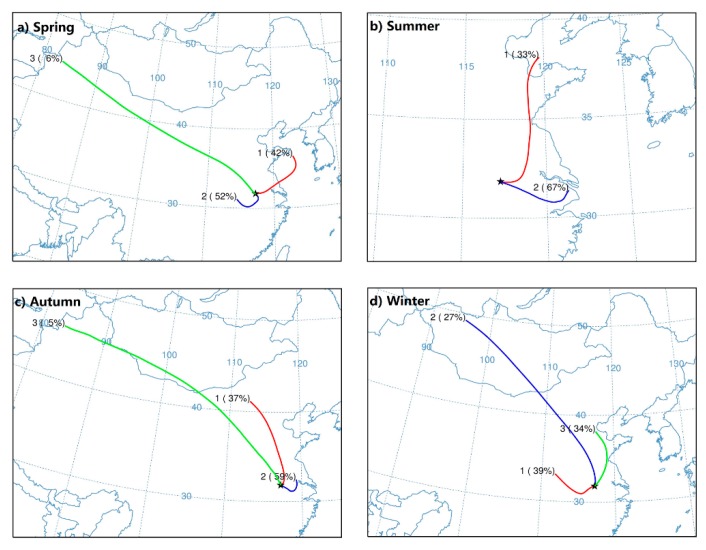
72 h airmass backward trajectories analysis during episode days (PM_2.5_ > 75 μg/m^3^) from December 2013 to November 2015.

**Figure 7:**
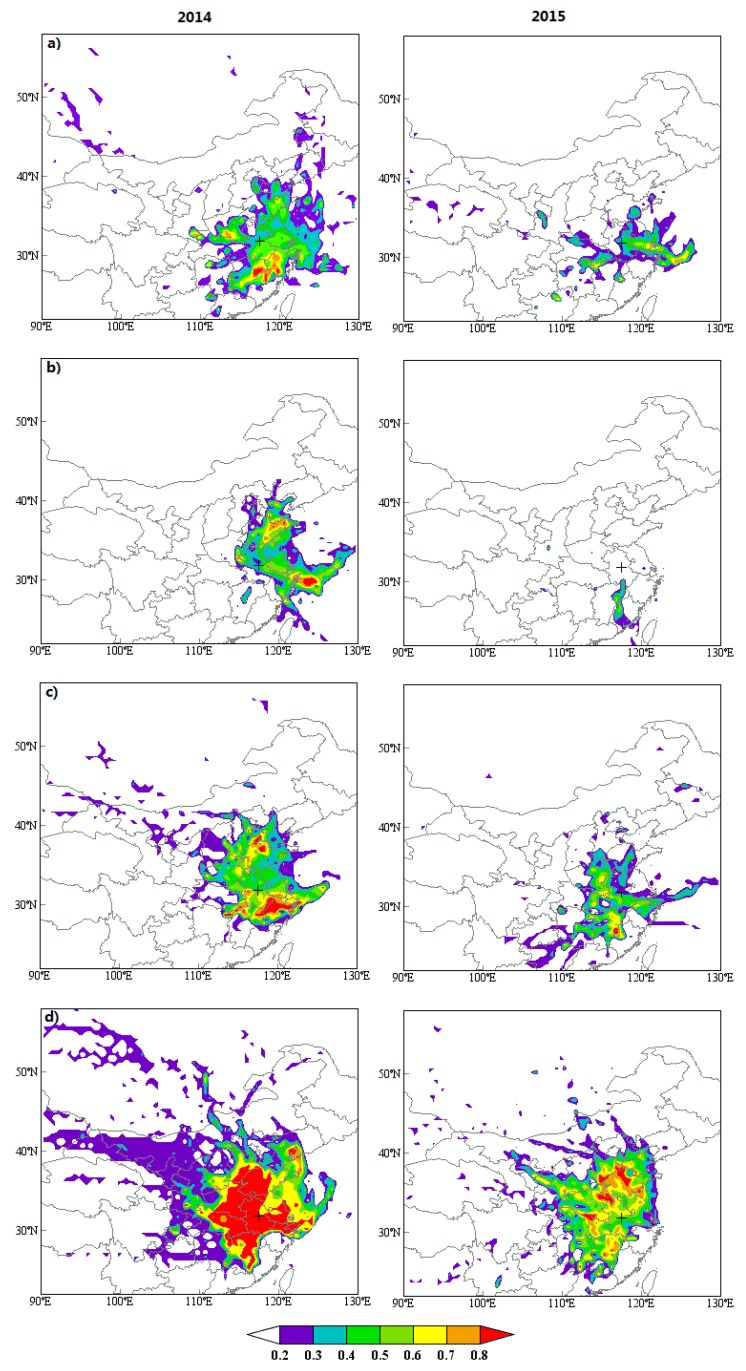
Seasonal potential source contribution function (PSCF) maps for PM_2.5_ at Hefei in 2014 and 2015: (**a**) spring, (**b**) summer, (**c**) autumn, (**d**) winter. The center of Hefei is marked by the cross and the PSCF values are displayed in color.

**Figure 8:**
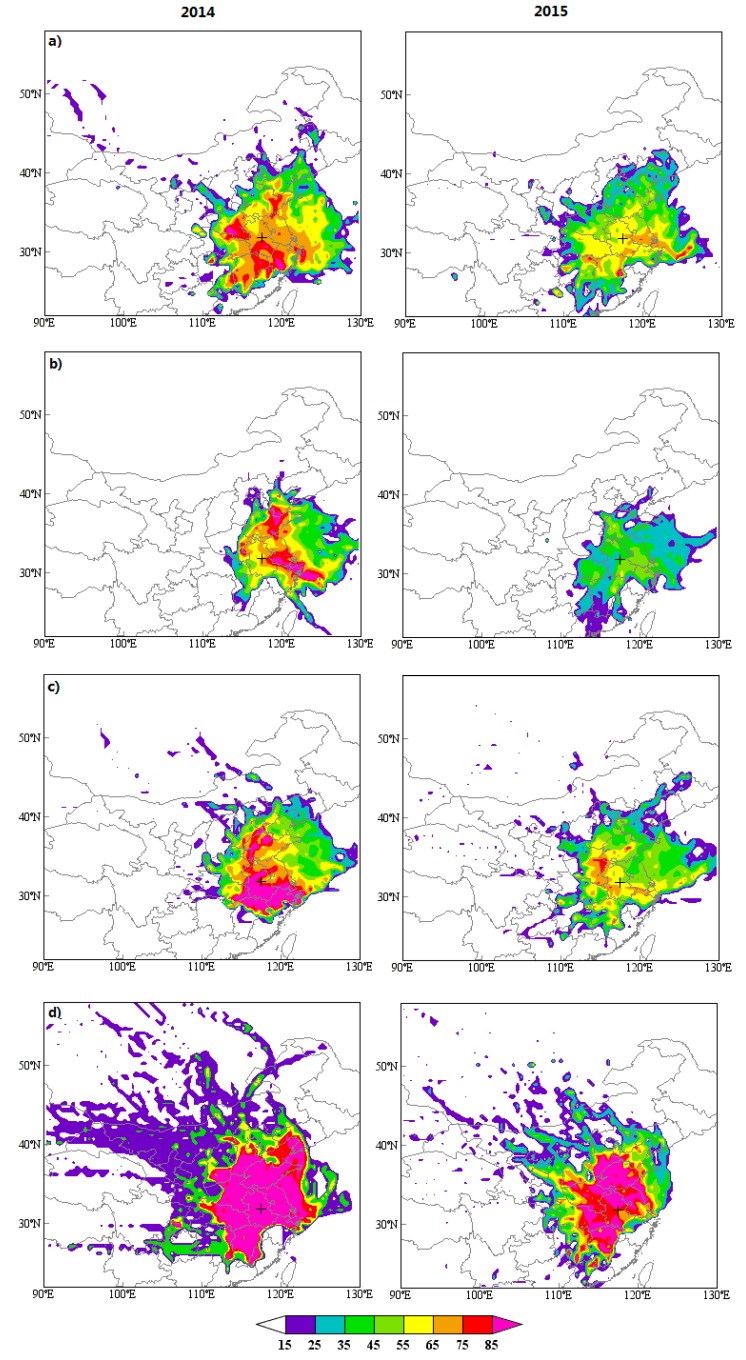
Seasonal concentration-weighted trajectory (CWT) maps for PM_2.5_ at Hefei in 2014 and 2015: (**a**) spring, (**b**) summer, (**c**) autumn, (**d**) winter. The center of Hefei is marked by the cross and the CWT values are displayed in color.

To assess local emission and regional transport of pollutants on air quality, the 72 h back trajectories from the central location of Hefei are clustered during PM_2.5_ episode days (Figure 6). PSCF and CWT models are conducted to make further efforts to reveal the potential source-areas and their relative contribution to the receptor site (Figure 7 and Figure 8). The topographical feature of Hefei signifies that it is easily affected by the polluted atmosphere that originated from its adjacent regions.

During springtime, the wind rose demonstrates that the prevailing wind is from the east (northeast, east, and southeast) (Supplementary Figure S3 and Supplementary Figure S4). The pollutants mainly come from local emissions (Cluster 2: 52%) and shorter-distance transport from YRD to the east (Cluster 1: 42%) region. Additionally, the frequency of dust weather in most parts of northern China is relatively high in spring, and air masses from these places carry the aerosol particles to Hefei and make the pollution in this area worse (Cluster 3: 6%). As displayed in Figure 7 and Figure 8, high PSCF/CWT to PM_2.5_ concentrations for Hefei is observed in south regions of Anhui and Jiangsu, southwest of Zhejiang, and northeast of Jiangxi with a PSCF > 0.5 and CWT > 75 μg/m^3^.

Summer is the best time of year for air quality, with mean PM_2.5_ concentrations of 69 μg/m^3^ (41 μg/m^3^) and attainment percentage of 67% (96%) during 2014 (2015). During summer, for one thing, PM emissions and secondary aerosol precursors are weak; for another, air masses are primarily derived from relatively clean regions under prevailing east wind (Supplementary Figures S3 and S4). Especially, air masses originating from the sea carry abundant water vapor and are beneficial to the formation of summer rainfall, and the accumulation of seasonal concentration of pollutants like PM_2.5_ is difficult during this period. Two clusters are identified during summer episode days. Cluster 1-Northest, accounting for 33% of air masses, stems from Bohai Bay and travels across middle of Shandong and North Jiangsu Province before reaching Hefei. Cluster 2-East is derived from Shanghai, across North Zhejiang and South Jiangsu Province before arriving at Hefei, accounting for 67% of total trajectories. The East Sea, north of Shandong Peninsula, and southwest of Jiangsu are identified as major potential sources areas of ambient PM_2.5_ by PSCF/CWT models. Furthermore, as compared to other seasons, both PSCF and CWT values are obviously lower in summer.

During autumn-time, major trajectories are divided into three groups by clusters analysis, accounting for 37% (Cluster 1), 59% (Cluster 2), and 5% (Cluster 3) of the total trajectories, respectively. Cluster 1 originates from the North China Plain (NCP). Cluster 2 begins in the YRD region, accounting for the largest percentage with features of short-distance air transport. Cluster 3 starts from Xinjiang, and passes through Inner Mongolia, Shanxi, Henan Province, and then reaches Hefei, which clearly indicates an extremely longer transport pathway in autumn. PSCF/CWT models identify that the south of Anhui and Jiangsu, north of Zhejiang, and northwest of Shandong are major potential sources areas of PM_2.5_.

Greater artificial release of PM_2.5_ is found in wintertime, and its average concentration is 141.2 ug/m^3^ (85 ug/m^3^), with only 22% (50%) attainment in 2014 (2015). A northwest pollution source with long-distance transmission is also observed for winter, but it is larger than that for autumn with accounting for approximately 27% of air masses. Local emissions from industry and fossil fuel and short-distance transmission from serious polluted NCP and YRD regions are the primary cause of the high levels of PM_2.5_, accounting for 39% and 34%, respectively. As illustrated in Figure 7 and Figure 8, the contribution from local emissions is found to be more striking for winter than the other three seasons, covering all the cities in Anhui Province. Particularly, high PSCF/CWT peaks, located in the south of Jiangsu and northwest of Shandong in autumn, spread to the whole Jiangsu and Shandong and most areas of Jiangxi, Henan, and Hubei Province, revealing that cross-boundary transport is crucial to PM_2.5_ pollution. Hefei also has a weak contribution to PM_2.5_ from distant Inner Mongolia and Xinjiang to the north with PSCF values basically between 0.2 and 0.5 and CWT between 25 and 75 μg/m^3^. Particularly, the East China Sea, the Yellow Sea, and the adjacent coastal region are potential sources of PM_2.5_.

In brief, the potential sources explicate obvious seasonal variation characteristics, and joint pollution control and prevention measurements need to further improve and implement on clean air in Hefei due to the impact of cross-boundary transportation.

### 3.7. Health Burdens Attributed to PM_2.5_ Exposure

**Figure 9:**
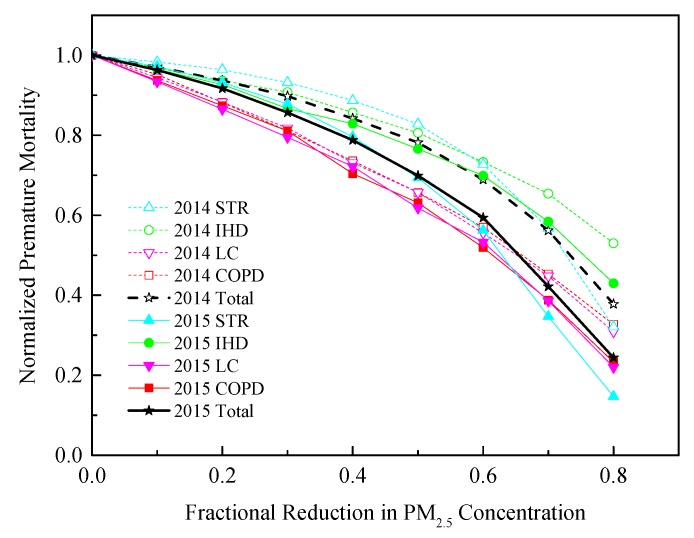
The avoidable premature mortality by cutting down PM_2.5_ concentrations to different levels.

Considering that PM_2.5_ is the trigger of some disease, the IER function, developed for the Global Burden of Disease Study, is used to produce a reasonable prediction of the relative risks of different diseases. The attributable fractions (AFs) of the ΔMort attributable to PM_2.5_ exposure in Hefei are 48% (44%), 32% (29%), 32% (26%), and 25% (21%) for STR, IHD, LC, and COPD in 2014 (2015), respectively (Supplementary Table S1). Similar to previous studies, a particularly strong correlation is revealed between excess STR mortality and PM_2.5_ exposure [48,49,50,51]. With reducing PM_2.5_ concentrations, the avoidable ΔMort caused by different diseases decreases correspondingly (Figure 9). The ΔMort reduction rate caused by LC and COPD is higher than STR and IHD, suggesting the benefits of reducing PM_2.5_ for respiratory diseases are higher than those for cardiovascular disease. Regardless of population growth in 2014, a 60% reduction of PM_2.5_ will result in 27%, 27%, 44%, and 43% ΔMort reductions for STR, IHD, LC, and COPD, respectively. The corresponding ΔMort reductions are 44%, 30%, 47%, and 48% relative to the 2015 level. PM_2.5_ concentrations need to be reduced by 63%, 80%, 65%, and 65% from the 2014 level to achieve a 50% reduction in STR, IHD, LC, and COPD-caused ΔMorts, and 63% 75%, 62%, and 60% relative to the 2015 level, respectively. A total of 50% ΔMort reduction requires an average of 72% (65%) reduction of PM_2.5_ in 2014 (2015), respectively.

**Figure 10:**
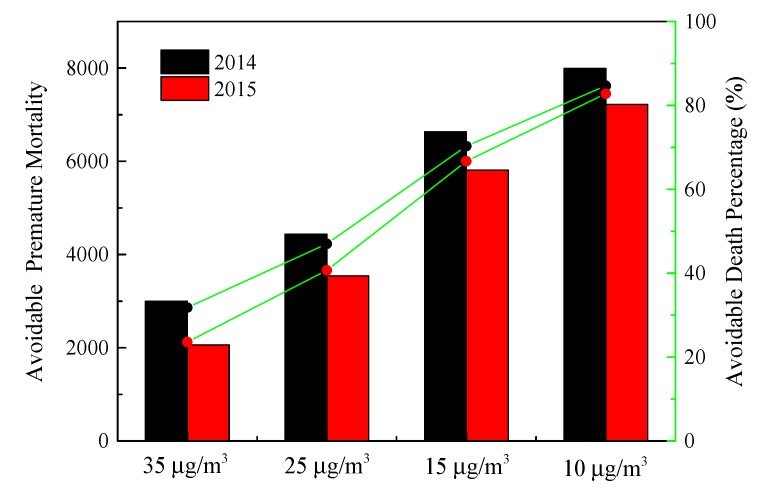
Potentially avoidable premature mortality and associated percentage from cutting down PM_2.5_ concentrations to four standards.

The potential avoidable ΔMort in Hefei due to PM_2.5_ exposure will be reduced by 32% from 9.44 to 6.44 thousand mortalities for situations in which ambient PM_2.5_ concentrations in 2014 meet the current CAAQS grade II standard. The evaluated avoidable death percentage will be 47%, 70%, and 85% when further reducing the PM_2.5_ concentrations to 25 μg/m^3^ (WHO IT2), 15 μg/m^3^ (WHO IT3), and 10 μg/m^3^ (WHO AQG), respectively (Figure 10). In 2015, the total ΔMort is about 8.53 thousand, and the mortality benefits will be 24%, 41%, 67%, and 83% of the excess mortalities attributable to PM_2.5_ if the PM_2.5_ concentrations are to meet the aforementioned four levels, respectively. The health burdens associated with PM_2.5_ in Hefei are still rigorous, much higher health benefits could be achieved if adopting more stringent WHO guidelines such as WHO IT3.

## 4. Conclusions

In this study, recently released air quality data with high spatial–temporal resolution are used to investigate the air pollution characteristics, influence factor, and associated health burden in the city of Hefei, central China. The annual PM_2.5_ (PM_10_) concentrations during 2014 and 2015 are 89.1 ± 59.4 µg/m^3^ (118.9 ± 66.8 µg/m^3^) and 61.6 ± 32.2 µg/m^3^ (91.3 ± 40.9 µg/m^3^), respectively, which exceed the CAAQS II standards. By comparison, trace gases (NO_2_, CO, SO_2_, and O_3_) concentrations are consistently below Grade-II limit. The Monthly (diurnal) variations of the pollutants with the exception of O_3_ present “U” (flat “M”) shape, whereas the O_3_ appears completely adverse trend pattern. The highest PM_2.5_ concentrations occur in cold seasons (January and December) as a result of synchronous control by emission from additional local heating and meteorological factors. PM_2.5_ is the major factor causing air pollution, and no gas species are found as major pollutants. Among 10 sites in Hefei, sites clustered in old districts have the highest pollution levels, followed by urban periphery sites, while new districts have the cleanest air. Higher ratios between PM_2.5_ and PM_10_ and CO for haze episode days than that for non-episode days reveal that the formation of secondary aerosols aggravates air pollution. According to the results of backward trajectory calculations, the ambient pollutant concentrations in Hefei tend to be influenced not only by air masses originated locally but also by air masses with short-distance and long-distance regional transport from adjacent provinces (especially Jiangsu, Hebei, Zhejiang, and Shandong) and remote region (Mongolia and Xinjiang), respectively. Based on the PSCF/CWT analysis, the potential sources explicate distinct seasonal variations. The common high-potential source regions of PM_2.5_ are located in most areas of Anhui, southern Jiangsu, northern Zhejiang, and western Shandong since these areas are the most important industrial base in China with dense population, rising energy consumption, as well as affected by higher pollutant emissions. Supposing that the PM_2.5_ concentrations are to meet the CAAQS grade II, the WHO IT-2, IT-3, and AQG, it will be possible to achieve mortality benefits of 32%, 47%, 70%, and 85% of the total premature mortalities (9.44 thousand) in 2014, and 24%, 41%, 67%, and 83% of the total premature mortalities (8.53 thousand) in 2015, respectively. This work suggests that cross-regional control measures and adopting more stringent standards are crucial to improve air quality in the region of urban central China.

## Figures and Tables

**Table 1:** Summary of air quality index (AQI) and the concentrations of six criteria air pollutants with standard deviations in Hefei (units are µg/m^3^ for PM_2.5_, PM_10_, NO_2_, SO_2,_ and O_3_, and mg/m^3^ for CO).

	Year	AQI	PM_2.5_	PM_10_	SO_2_	NO_2_	CO	O_3_
**Annual**	2014	118.1 ± 68.6	89.1 ± 59.4	118.9 ± 66.8	30.2 ± 10.6	28.2 ± 11.1	1.1 ± 0.5	50.4 ± 18.8
	2015	86.6 ± 38.1	61.6 ± 32.2	91.3 ± 40.9	17.3 ± 8.8	31.0 ± 10.6	1.0 ± 0.3	64.8 ± 29.4
**Spring**	2014	100.8 ± 29.1	73.5 ± 22.7	112.6 ± 52.7	18.2 ± 5.8	29.8 ± 7.4	0.9 ± 0.2	56.9 ± 14.9
	2015	86.9 ± 26.7	61.2 ± 23.2	96.7 ± 33.4	19.2 ± 6.3	26.9 ± 8.8	1.0 ± 0.3	61.1 ± 18.1
**Summer**	2014	93.6 ± 45.0	68.7 ± 38.2	101.7 ± 47.9	14.4 ± 6.4	20.5 ± 6.0	1.0 ± 0.3	63.9 ± 21.0
	2015	63.6 ± 21.3	41.3 ± 19.1	69.8 ± 28.2	9.5 ± 4.0	27.7 ± 7.0	0.9 ± 0.2	87.0 ± 24.6
**Autumn**	2014	101.6 ± 54.9	74.8 ± 46.9	112.0 ± 60.9	18.1 ± 6.1	25.9 ± 9.3	1.0 ± 0.3	42.0 ± 15.6
	2015	83.5 ± 33.1	59.2 ± 27.6	90.7 ± 39.3	13.6 ± 5.1	37.9 ± 13.0	1.1 ± 0.3	75.3 ± 30.9
**Winter**	2014	178.3 ± 92.2	141.2 ± 81.6	150.4 ± 89.4	30.7 ± 14.0	37.0 ± 13.6	1.5 ± 0.7	38.5 ± 9.3
	2015	113.0 ± 48.9	84.5 ± 39.9	108.8 ± 50.2	27.1 ± 7.4	31.9 ± 9.3	1.1 ± 0.3	35.3 ± 10.6

**Table 2:** Statistics of general meteorological parameters at Hefei *.

Meteorological Parameters	Year	Total	Spring	Summer	Autumn	Winter	Non-Episode	Episode
T (℃)	2014	16.5	17.1	26.3	18.0	4.4	18.6	13.5
	2015	16.9	16.8	26.4	17.8	5.5		
RH (%)	2014	76	71	81	78	73	76	73
	2015	74	72	80	77	67		
WS (m/s)	2014	1.9	2.2	1.8	1.9	1.9	2.1	1.8
	2015	2.0	2.0	2.1	1.9	2.0		
Pre (mm)	2014	1237	298	546	247	146	2200	334
	2015	1297	325	673	196	103		

* Note: mean values for air temperature (T), relative humidity (RH), wind speed (WS), accumulated value of precipitation (Pre).

**Table 3:** Average diurnal concentrations of six criteria air pollutants at ten stations in Hefei during 2015 (units are µg/m^3^ for PM_2.5_, PM_10_, SO_2_, NO_2_, and O_3_, and mg/m^3^ for CO).

Station	Description	PM_2.5_	PM_10_	SO_2_	NO_2_	CO	O_3_
*old district*							
YR	Yangtze River Road	64.6	106.6	21.0	35.6	1.2	71.8
YH	Yaohai District	66.3	99.1	13.7	31.2	1.1	77.0
SL	Sanlijie	61.8	96.7	14.1	58.4	1.1	94.7
AV	Amber Villa	64.9	90.9	18.1	26.2	0.9	79.9
*urban periphery*							
LY	Luyang District	65.9	99.3	18.4	15.3	1.0	40.9
DP	Dongpu Reservoir	60.5	90.9	20.0	26.9	0.7	92.6
BH	Baohe District	57.5	88.7	18.8	26.3	1.0	52.3
PP	Pearl Plaza	56.9	88.9	20.3	55.8	1.0	102.2
*new district*							
HT	High-tech Zone	60.8	87.7	16.6	17.5	1.0	38.1
BN	Binhu New Zone	58.9	90.6	11.0	19.3	0.9	39.4

**Table 4:** The Pearson Correlations between six pollutants and meteorological elements (RH: relative humidity; T: temperature; WS: wind speed) in the non-episode days (cells above the diagonal) and episode days (cells below the diagonal).

	RH	T	WS	PM_2.5_	PM_10_	SO_2_	CO	NO_2_	O_3_
**RH**	−	0.13	−0.09	−0.18	−0.53	−0.47	0.10	−0.31	−0.18
**T**	0.16	−	−0.08	−0.20	0.12	−0.57	0.02	−0.06	0.58
**WS**	−0.18	0.00	−	−0.29	−0.25	−0.02	−0.38	−0.36	−0.13
**PM_2.5_**	−0.06	−0.29	−0.08	−	0.68	0.43	0.57	0.36	0.02
**PM_10_**	−0.16	0.02	−0.15	0.81	−	0.39	0.40	0.48	0.24
**SO_2_**	−0.43	−0.51	−0.06	0.52	0.45	−	0.25	0.31	−0.33
**CO**	0.15	−0.27	−0.26	0.82	0.67	0.48	−	0.57	0.12
**NO_2_**	−0.28	−0.28	−0.40	0.36	0.44	0.50	0.54	−	0.28
**O_3_**	−0.09	0.68	−0.03	−0.14	0.10	−0.29	−0.17	−0.02	−

## Supplementary Materials

The following are available online at https://www.mdpi.com/1660-4601/17/1/229/s1: Data and Methods, Figure S1: Diurnal variations of SO_2_ (upper) and CO (down), Figure S2: The seasonal variations of PM_2.5_/PM_10_ ratios and PM_2.5_/CO ratios, Figure S3: The relationship between percent frequency and wind speed, direction for seasonal distribution in 2014 at Hefei, Figure S4: The relationship between percent frequency and wind speed, direction for seasonal distribution in 2015 at Hefei, Table S1: The attributable fractions due to PM_2.5_ for STR, IHD, LC, and COPD.
