# Supplementary material for: Spatiotemporal Variations and Factors of Air Quality in Urban Central China during 2013–2015
Source: Int J Environ Res Public Health. 2019 Dec 28;17(1):229. doi: 10.3390/ijerph17010229 (PMC6981523; doi:10.3390/ijerph17010229)
Supplement: Supplementary file 1 [file ijerph-17-00229-s001.pdf]

# Spatiotemporal variations and factors of air quality in urban central China during 2013–2015

# Supplementary data

Mao Mao [1], Xiaolin Zhang [1,*], Yamei Shao [1], Yan Yin [1],

[1] Key Laboratory for Aerosol-Cloud-Precipitation of China Meteorological Administration, School of Atmospheric Physics, Nanjing University of Information Science & Technology, Nanjing 210044, China

* Correspondence: xlnzhang@nuist.edu.cn

# Data and Methods

## Data

Designed as a mix of nine urban sites and one background sites for the point layout of ambient air quality monitoring, presently there are ten national automated monitoring sites in Hefei. Monitoring systems were installed and used to measure the pollutant concentrations according to China Environmental Protection Standards (HJ655-2013). The mass concentrations of $PM_{2.5}$ and $PM_{10}$ were measured using the Tapered Element Oscillating Microbalance analyzers, and levels of $SO_2$, $NO_2$, $O_3$ and CO were measured using the using of the ultraviolet fluorescence method, the chemiluminescence method, the UV-spectrophotometry method, and the non-dispersive infrared absorption method (or the gas filter correlation infrared absorption method), respectively. The diurnal concentrations of each pollutant were calculated only when there were more than 16 h valid data. To better study the temporal changes of pollutants, the corresponding data are divided into four seasons: spring (March–May), summer (June–August), autumn (September–November) and winter (December–February).

## Methodology

***Hybrid Single Particle Lagrange Integrated Trajectory (HYSPLIT) model***

HYSPLIT-4 model was developed in the Air Resources Laboratory (ARL) of the National Oceanic and Atmospheric Administration (NOAA). Corresponding meteorological data with a 1° × 1° grid used in the HYSPLIT-4 model was acquired from the Global Data Assimilation System (GDAS) (ftp://arlftp.arlhq.noaa.gov/pub/archives/gdas1/). The backward trajectories were run four times per day at a starting times of 0:00, 06:00, 12:00, and 18:00 UTC (8:00, 14:00, 20:00, and 02:00 LT, respectively) with starting height of 500 m above ground level (agl). In order to divide the seasonal trajectories into distinct transport groups, cluster analysis was carried out on the basis of the hierarchical clustering method [1].

***Potential source contribution function (PSCF)***

The zone of concern is divided into small equal grid cells ($i \times j$). The PSCF value in the $ij^{th}$ cell represents the probability of air parcel residence time and is expressed as:

$$PSCF_{ij} = \frac{m_{ij}}{n_{ij}} \tag{1}$$

where $i$ and $j$ denote the latitude and longitude, $n_{ij}$ is on behalf of the total number of endpoints that fall in the $ij^{\text{th}}$ cell, and $m_{ij}$ represents the number of endpoints in the same cell associated with samples for which the monitored mass concentration of pollutant exceeds the criterion value in the $ij^{\text{th}}$ cell. The GB3095-2012 $PM_{2.5}$ guideline value for daily average of 75 μg/m$^3$ is used as the criterion-value. To better reflect the uncertainty in cells, the PSCF values should be multiplied by an arbitrary weighting function $W(n_{ij})$ when $n_{ij}$ is lower than three times of average number of trajectory endpoints ($n_{ave}$) in each cell [2,3], and the weight PSCF (WPSCF) is described as follows:

$$WPSCF_{ij} = \frac{m_{ij}}{n_{ij}} \times W(n_{ij}) \tag{2}$$

$$W(n_{ij}) = \begin{cases} 1.00, & 3n_{ave} < n_{ij} \\ 0.70, & 1.5n_{ave} < n_{ij} \leq 3n_{ave} \\ 0.40, & n_{ave} < n_{ij} \leq 1.5n_{ave} \\ 0.20, & n_{ij} \leq n_{ave} \end{cases} \tag{3}$$

***Concentration-weighted trajectory (CWT) model***

Since PSCF just reflects the proportion of polluted trajectories in a cell grid, which is impossible to distinguish the contribution to the pollution levels of the target object for those who have the same PSCF value, CWT model is applied to weight trajectories with related $PM_{2.5}$ concentrations for better unraveling above limitation and calculating the relative contribution of different sources-areas. The geographical field is divided into grid cells representing an area of 0.5 ° × 0.5 °. The CWT is defined as follows:

$$C_{ij} = \frac{\sum_{h=1}^{M} C_h \times \tau_{ijh}}{\sum_{h=1}^{M} \tau_{ijh}} \times W(n_{ij}) \tag{4}$$

Where $C_{ij}$ is designed as the average weight concentration of the trajectory $h$ in the $ij^{th}$ cell, $h$ represents the index of the trajectory, $M$ represents the total number of trajectories, $C_h$ denotes $PM_{2.5}$ concentrations in the trajectory $h$ through $ij^{\text{th}}$ cell, $\tau_{ijh}$ is the time that trajectory $h$ resides in the $ij^{\text{th}}$ cell ($i$,

*j*). The $W(n_{ij})$ used in PSCF also is utilized to CWT method for reducing the uncertainty in cells.

The study domain is in the range of 20–60 °N and 90–130 °E, which includes more than 95% of areas covered by all the paths. The total number of endpoints in each season is about 51840, and the area covered by the trajectories is divided into 6400 grid cells (0.5 ° × 0.5°). Hence, each cell owns an average of 9 trajectory endpoints, that is, the $n_{ave}$ is equal to 9. Both PSCF and CWT analysis are run by the MeteoInfo soltware-TrajStat Plugin.

***Integrated exposure–response (IER) model***

This model is based on the assumption that the cause-specific mortality rates are independent of $PM_{2.5}$ concentrations. The relative risk (RR) for a specific disease due to $PM_{2.5}$ exposure is the ratio of the probability of an event occurring in an exposed group to the probability of the event occurring in a comparison, nonexposed group [4,5]. Adult STR (stroke, including ischemic and hemorrhagic stroke), Ischemic heart disease (IHD), lung cancer (LC) and chronic obstructive pulmonary disease (COPD), ranking top ten in the international classification, are selected as the causes of mortality. The mortality burdens attributable to ambient $PM_{2.5}$ exposure in 2014 and 2015 are calculated using the disease-specific mortality and Hefei population data. The RR of several causes of premature mortality (*Δ*Mort) among adults including STR, IHD, LC, and COPD, are estimated according to the IER functions.

The RR for each disease is calculated based on equation (5):

$$RR(C) = \begin{cases} 1 + \alpha\,(1 - e^{-\gamma (C - C_0)^{\delta}}), & C \geq C_0 \\ 1, & C < C_0 \end{cases} \tag{5}$$

where $C$ is the annual $PM_{2.5}$ exposure concentration (μg/m$^3$); $C_0$ is the endpoint-specific theoretical minimum-risk concentration of $PM_{2.5}$; α, δ and γ are parameters used to describe the shape of C–RR curves. The population attributable fraction (AF), representing the fraction of mortality for a specific disease due to $PM_{2.5}$ exposure, is calculated by RR factors through the formula (6):

$$AF = (RR - 1)/RR \tag{6}$$

The AFs are estimated with the annual average $PM_{2.5}$ concentrations in 2014 and 2015 for four diseases. Formula (7) is used to calculate the *Δ*Mort for a specific disease attributable to $PM_{2.5}$ exposure

[6,7]. $y_0$ is the baseline mortality rate caused by a specific disease, and Pop is the total population exposed to $PM_{2.5}$ in Hefei. The population data of Hefei in 2014 and 2015 are obtained from Hefei Statistics Yearbook.

$$\Delta Mort = y_0 \times AF \times Pop \quad (7)$$

## References


[1] Draxler, R.R., Hess, G.D., 1998. An overview of the HYSPLIT 4 modeling system for trajectories dispersion and deposition. Aust. Meteorol. Mag. 47, 295–308.

[2] Polissar, A.V., Hopke, P,K., Poirot, R.L., 2001. Atmospheric aerosol over Vermont: chemical composition and sources. Environ. Sci. Technol. 35, 4604–4621.

[3] Dimitriou, K., Kassomenos, P., 2015. Three year study of tropospheric ozone with back trajectories at a metropolitan and a medium scale urban area in Greece. Sci. Total Environ. 502, 493–501.

[4] Lelieveld, J., Evans, J.S., Fnais, M., Giannadaki, D., Pozzer, A., 2015. The contribution of outdoor air pollution sources to premature mortality on a global scale. Nature 525, 367–371.

[5] Hu, J., Huang, L., Chen, M., Liao, H., Zhang, H., Wang, S., Zhang, Q., Ying, Q. 2017. Premature mortality attributable to particulate matter in China: Source contributions and responses to reductions Environ. Sci. Technol. 51, 9950–9959.

[6] Anenberg, S.C., Horowitz, L.W., Tong, D.Q., West, J.J., 2010. An estimate of the global burden of anthropogenic ozone and fine particulate matter on premature human mortality using atmospheric modeling. Environ. Health. Perspect. 118, 1189–1195.

[7] Lelieveld, J., Barlas, C., Giannadaki, D., Pozzer, A., 2013. Model calculated global, regional and megacity premature mortality due to air pollution. Atmos. Chem. Phys. 13, 7023–7037.

**Table S1** The attributable fractions due to $PM_{2.5}$ for STR, IHD, LC and COPD.

| | $PM_{2.5}$ | Attributable Fractions (AFs) (%) | | | |
|---|---|---|---|---|---|
| | (μg/m$^3$) | STR | IHD | LC | COPD |
| 2014 | 89 | 48 | 32 | 32 | 25 |
| 2015 | 61 | 44 | 29 | 26 | 21 |
| CAAQS grade II | 35 | 34 | 23 | 18 | 15 |
| WHO IT−2 | 25 | 25 | 20 | 14 | 11 |
| WHO IT−3 | 15 | 11 | 15 | 8 | 7 |
| WHO AQG | 10 | 4 | 10 | 4 | 3 |

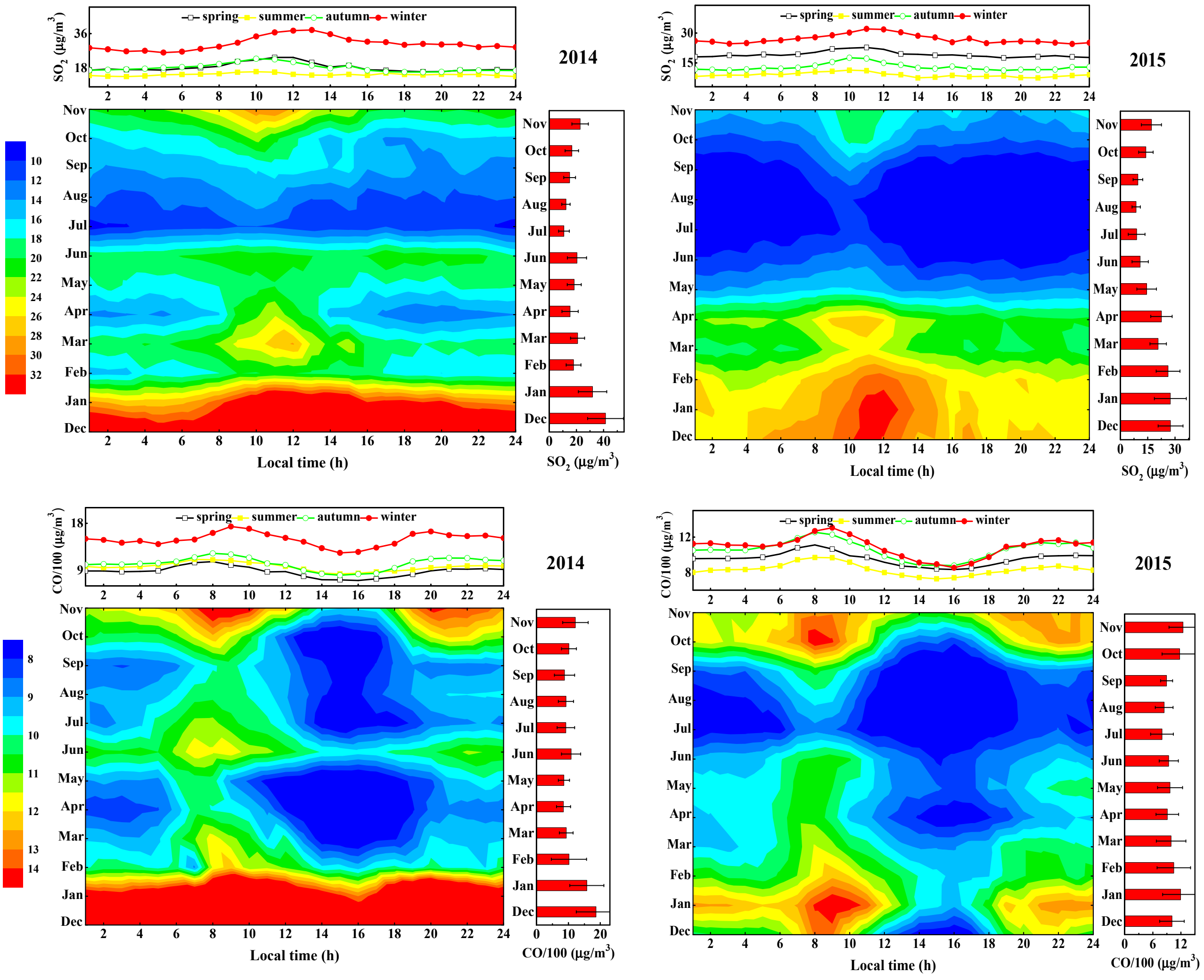


**Fig. S1.** Diurnal variations of $SO_2$ (upper) and CO (down).

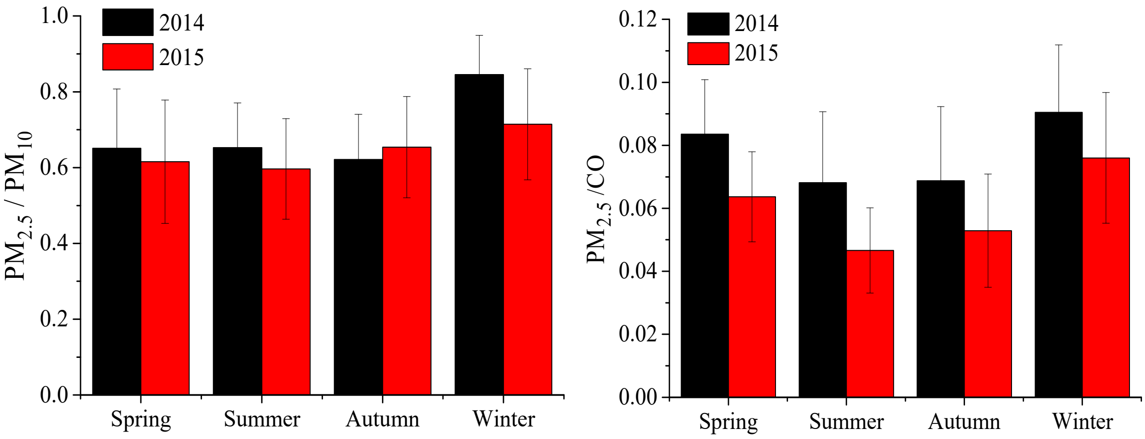


**Fig. S2.** The seasonal variations of $PM_{2.5}/PM_{10}$ ratios and $PM_{2.5}/CO$ ratios.

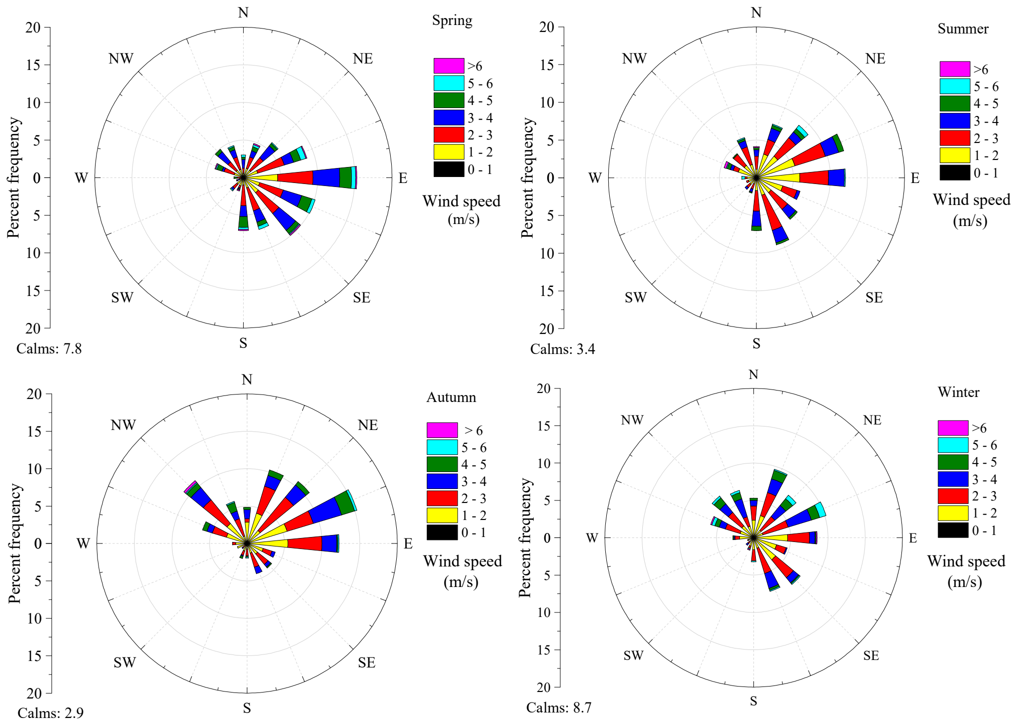


**Fig. S3.** The relationship between percent frequency and wind speed, direction for seasonal distribution in 2014 at Hefei.

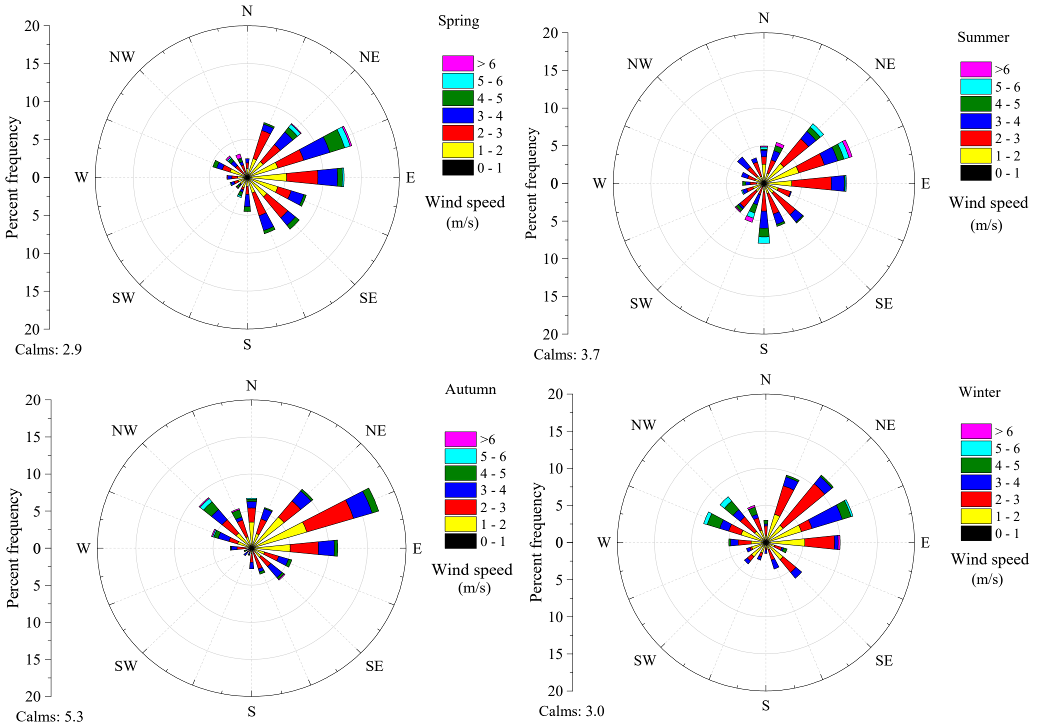


**Fig. S4.** The relationship between percent frequency and wind speed, direction for seasonal distribution in 2015 at Hefei.
